# Predictors of Loneliness among Homeless Individuals in Germany during the COVID-19 Pandemic

**DOI:** 10.3390/ijerph191912718

**Published:** 2022-10-05

**Authors:** Katharina Dost, Fabian Heinrich, Wiebke Graf, Anna Brennecke, Veronika Kowalski, Anna Leider, Anika Kraus, Victoria van Rüth, Benjamin Ondruschka, Klaus Püschel, Hans-Helmut König, Franziska Bertram, André Hajek

**Affiliations:** 1 Institute of Legal Medicine, University Medical Center Hamburg-Eppendorf, 22529 Hamburg, Germany; 2 Department of Health Economics and Health Services Research, University Medical Center Hamburg-Eppendorf, 20246 Hamburg, Germany

**Keywords:** homelessness, loneliness, social isolation, social exclusion, COVID-19, SARS-CoV-2

## Abstract

Purpose: The aim of the study was to identify the frequency of loneliness and to examine the factors associated with loneliness among homeless individuals in Germany during the COVID-19 pandemic. Methods: Data were taken from the ‘national survey on the psychiatric and somatic health of homeless individuals during the COVID-19 pandemic’. The data collection took place from 26th July to 17th September 2021 (the analytical sample included *n* = 491 observations). The well-established UCLA-3 tool was used to quantify loneliness. Independent variables included sex, age, marital status, the existence of children and pets, level of education, country of origin, duration of homelessness, alcohol and drug consumption, mental health concerns and concerns regarding COVID-19 illness. Multiple logistic regressions were used to examine the predictors of loneliness. Results: The frequency of loneliness was 41.7% for the total sample. Multiple logistic regression analysis stratified by gender showed that a higher likelihood of loneliness was associated with being born in Germany, being middle aged (40 to 49 years compared to 18 to 29 years), having mental health problems and a short period of homelessness (1 month compared to longer periods) among women. In men, a higher likelihood of loneliness was associated with a higher fear of COVID-19 and a short period of homelessness. Conclusions: Our study revealed a high frequency rate of loneliness among homeless individuals. The study results highlight the associations between some explanatory variables (i.e., the duration of homelessness and mental health problems). Identifying the factors associated with loneliness may help to adequately address the problems of homeless individuals at risk of loneliness. Longitudinal studies are required to confirm our findings.

## 1. Introduction

According to estimates, in 2018, about 678,000 people were homeless in Germany. Based on forecasts, the number of homeless people is still further increasing [1].

The homeless population has become increasingly vulnerable during the COVID-19 pandemic. More precisely, due to crowding in shelters, the use of congregate sleeping arrangements, limited access to hygienic supplies and some asymptomatic infections [2], the transmission of SARS-CoV-2 could increase. Furthermore, common methods used to control COVID-19 spreading in shelters (e.g., testing, contact tracing, physical distancing and restricting movement) are difficult to implement and control. Thus, infections could spread more quickly [3], and homeless individuals could potentially become super-spreaders [4]. In addition, homeless individuals are at risk of experiencing a more severe course of COVID-19 due to pre-existing conditions [5]. Compared to the general population, homeless individuals have a higher frequency of infectious and chronic diseases and higher mortality (e.g., below 65 years, the frequency of all-cause mortality is 5–10 times higher) [4,6]. Much of the excess mortality compared to the general population may be explained by increased exposure to risk factors, including excessive alcohol consumption, smoking, illicit drug use and mental health disorders, which often coexist [7] and accompany each other.

Due to the COVID-19 pandemic, loneliness (the negative experience arising from the perceived discrepancy between actual and desired social relations) [8] is a common and significant global threat [9]. According to Cacioppo and Hawkley, perceived social isolation (loneliness) is more closely related to the quality than the quantity of social interactions [10]. While loneliness is correlated with social isolation (i.e., lack of social activities), it is not measured as the same construct [11]. They also somewhat differ in their predictors and consequences. [12,13] Moreover, there may be differences in the predictors of loneliness betewen males and females (Maes et al., 2019; Takagi et al., 2020) [14,15,16]. Therefore, sex-stratified regressions appear to be important.

In the EU alone, the number of people feeling lonely doubled after the COVID-19 outbreak [17]. A survey of the German population in the late summer of 2021 revealed a frequency of loneliness of 83.4% [18]. Based on longitudinal data from multiple studies in several EU countries conducted over the initial lockdown period (March to July 2020), increased percentages were found in the United Kingdom, Denmark and France, while only 7% of respondents in the Netherlands reported high levels of loneliness [19].

Previous studies have already examined the predictors of loneliness in certain other populations. In particular, individuals in old age showed an increased risk of loneliness during the COVID-19 pandemic [20], as well as younger people and those with a history of mental illness, who reported the highest levels of loneliness, while women and those with previously diagnosed chronic illness also reported slightly higher levels of loneliness [19]. However, little is known about loneliness among homeless individuals at present [11,21]. Only the frequency of loneliness among homeless individuals in one city (Hamburg, the second largest city in Germany) during the first wave of the pandemic (May/June 2020) has been studied thus far. In total, 48.5% of participants felt lonely, and higher loneliness was associated with being male, being single and a higher perceived risk of contracting COVID-19 [22] and in the later stage of the pandemic (May–June 2020) in France, the frequency of loneliness among the homeless population was 37% [23]. Thus far, only a few studies have addressed the problem of loneliness among homeless individuals.

It is highly important to identify at-risk groups, with high levels of loneliness among the homeless individuals, as feelings of loneliness are increasingly recognized as a risk factors for worse physical and mental health, which in turn can affect mortality [24]. This has also increased interest in interventions to reduce chronic loneliness [25].

It is necessary to describe the pandemic situation in Germany at the time of data collection. At the beginning of the survey (July 2021), the incidence rate was 14.3 cases per 100,000 among the population per week. In the following weeks, the incidence rate increased significantly throughout Germany, rising much earlier and faster than the year before, which marked the beginning of the fourth wave. By the end of the study (September 2021) the 7-day incidence was 74.7 cases per 100,000 among the population per week.

Thereafter, the Delta variant became the most common coronavirus type in Germany. In order to prevent the further increase in the infection numbers, the countries were obligated to comply the 3G rule (access was permitted almost everywhere only for vaccinated, recovered or tested persons) from 23 August 2021 onwards [26]. By mid-September 2021, 67% of the German population had been vaccinated at least once, and 62% has been fully vaccinated (either received a required second vaccination or vaccinated with a vaccine that provides full vaccine protection even when the recipient is vaccinated once) [27].

Given the limited knowledge of this topic, the aim of this multicentric study was to further investigate loneliness and its predictors of loneliness among homeless individuals during a later stage of the pandemic and in other German metropolitan regions (i.e., end of July to mid-September 2021).

## 2. Materials and Methods

### 2.1. Sample

Cross-sectional data were taken from the ‘national survey on the mental and somatic health of homeless individuals during the COVID-19 pandemic—NAPSHI’. The study was conducted by means of questionnaire-based interviews, which took place at night shelters, women’s shelters, lodging houses, medical practices or drug counselling centers. In total, 761 individuals were recruited in the period from 26th July to 17th September 2021. Data were collected in four large German cities (Hamburg, Frankfurt am Main, Leipzig and Munich) and additionally in Mainz, Wiesbaden and Augsburg. An incentive of EUR 5 for 30 min of involvement was offered to all participants. The main inclusion criteria were an age of ≥18 years, no permanent residence for more than 7 days and informed consent to participate in the study. The main exclusion criterion was an existing pregnancy. In addition, we excluded individuals who did not wish to be informed of incidental findings that indicate a potentially life-threatening disease. Demographic, psychological and somatic health data were collected, a brief physical examination was conducted and a blood sample was taken. The self-developed German questionnaire used was translated by native speakers into different languages (English, Russian, Polish and Bulgarian). Depending on the level of language comprehension, the questionnaire was either filled out by the participants or with the help of a translator, mainly performed by native speakers (other participants or employees of the institutions). The analytical sample (descriptive analysis) equaled 634 individuals. In the final analytical sample (regression analysis), *n* = 496 individuals were included (due to missing values in the independent variables). Written informed consent was provided by all participants prior to the investigation. The study was approved by the Ethics Committee of the Hamburg Chamber of Physicians (No. PV7333).

### 2.2. Dependent Variable

Perceived loneliness was measured using the UCLA Loneliness Scale Version 3 (UCLA-3). The Three-Item Loneliness Scale is a commonly used measure with the benefits of favorable reliability and validity [28], consisting of the following items: “How often do you feel that you lack companionship?”; “How often do you feel left out?“; and “How often do you feel isolated from others?” Each item can be answered on a 3-point scale: 1 = “hardly ever”; 2 = “some of the time”; and 3 = “often” [29]. The scores for each question were summed to obtain a loneliness score ranging from 3 to 9. As suggested, an average of 3 to 5 is defined as “not lonely”, while higher sum scores of 6 and above are defined as loneliness [30].

### 2.3. Independent Variables

We selected our independent variables based on prior research and based on theoretical considerations. For example, in a systematic review, Dahlberg [31] showed that marital status is associated with loneliness. Another example, a systematic review by Kretzler et al., showed that pet ownership is important for loneliness [32].

In the regression analysis, (self-reported) sociodemographic variables were included as follows: age (in years), sex (male/female), marital status (married and living together, married but permanently separated from spouse, single, widowed or divorced), number of children, level of education (categorized as no graduation; school education, graduation from main/middle school, university qualification, vocational education, higher tertiary education with an university degree), country of origin (Germany/other countries) and duration of homelessness (in months, categorized into: >1 month, 2–12 months and >13 months). In addition, explanatory variables included (self-reported) lifestyle factors, such as the keeping of pets (yes/no), consumption of alcohol, as well as illicit drug use in the past year (never, once, twice a year, monthly, weekly, daily) and health-related factors, including mental health concerns (yes/no; individuals reported having been officially diagnosed by their physicians) and concerns about COVID-19 (self-reported: not at all, a bit, some, strongly). It is worth noting that concerns regarding COVID-19 were treated as a continuous variable for reasons of simplicity.

### 2.4. Statistical Analysis

First, frequency rates of loneliness (stratified by sex) were reported for several subgroups. A logistic regression model was used to identify the correlates of high loneliness levels. Statistical significance was defined as a *p*-value of *p* < 0.05 throughout all the tests. Analyses were performed using Stata 16.0 (StataCorp, College Station, TX, USA).

## 3. Results

The sample included a total number of 634 participants. The average age was 43.4 years (SD: 12.2 years, ranging from 18 to 80 years), and most of them were men (82.3%). The mean duration of homelessness was 43 months (SD: 67 months). About half of the participants (51.2%) were born in Germany. Most were single (66.9%), 47.6% had at least one child and 7.9% had a pet.

Frequency rates of loneliness are presented in Table 1. Among the total sample, the frequency of loneliness was 41.7% (*n* = 265; among men: 40.7%; among women: 47.8%).

The average loneliness score (measured continuously) was 5.2 (SD: 1.9) among the total sample. Among men, it was 5.1 (SD: 1.9), and it was 5.4 (SD: 2.0) among women. A high frequency of loneliness was mainly observed among middle-aged individuals (40 to 49 years; 45.6%; men: 44.8%; women: 50.0%), individuals born in Germany (47.0%; men: 46.3%; women: 51.4%) and individuals who had been homeless for only one month but no longer (57.1%; men: 51.4%; women: 75.0%), as well as individuals who reported to have been officially diagnosed with mental health problems by their physicians (56.3%; men: 53.3%; women: 65.8%). Further details are given in Table 1, as well as Supplementary Table S1, with correlation matrices for the continuous measurements (among the total sample).

Findings of logistic regression analyses are displayed in Table 2. In men, a higher likelihood of loneliness was associated with a higher fear of COVID-19 (OR: 1.34, 95% Cl: 1.06–1.70) and a lower duration of homelessness (2 to 12 months, OR: 0.38, 95% Cl: 0.16–0.91, compared to a duration of up to 1 month).

In the case of women, the logistic regression analysis revealed that a higher likelihood of loneliness was associated with older age (especially from 40 to 49 years, OR: 37.43, 95% Cl: 1.58–885.63, compared to an age ranging from 18 to 29 years), a lower duration of homelessness (e.g., 2 to 12 months, OR: 0.02, 95% Cl: 0.00–0.26, compared to a duration of up to 1 month), being born in Germany (OR: 8.57, 95% Cl: 1.11–66.52, compared to being born abroad) and existing mental problems (OR: 5.89, 95% Cl: 1.31–26.39, compared to the absence of psychic problems).

## 4. Discussion

Loneliness is not only a feeling but also a public health problem. As already mentioned, it is associated with an increased risk of mental as well as physical illness. Feeling lonely, especially over a long period of time, can negatively impact mental health (e.g., greater depression and suicidal ideation) and can lead to serious health problems (e.g., impacts on cardiovascular health, morbidity and mortality), mainly if left untreated [33]. Many predictors of loneliness have already been studied in detail among different target groups. Systematic reviews demonstrated a positive association between age and loneliness [34]. In particular, the elderly group experienced increasing loneliness, related to the predictors of being unmarried/unpartnered and having lost a partner or having a limited social network [31,35].

The aim of this multicentric study was to investigate the predictors of loneliness in a later stage of the COVID-19 pandemic, mainly during third to fourth wave. Overall, 41.7% of homeless individuals in our multicenter study experienced loneliness. This is one of the first studies determining the frequency and the correlates of loneliness among homeless individuals and, therefore, it markedly extends our current knowledge.

The frequency of loneliness is highly dependent on the time period and the population observed. As data on loneliness among homeless individuals at the time of the COVID-19 pandemic are scarce, it is difficult to compare the frequency of loneliness identified in our study with previous findings.

In general, a finding of a stable or slightly decreasing frequency of loneliness during the pandemic is in line with data from other studies, using data from the general population [36,37,38].

A direct comparison between the general population and homeless individuals is rather difficult. However, we can assume that homeless individuals also experienced loneliness, social exclusion or separation from family members prior to the pandemic, and that adverse circumstances have contributed to individual coping abilities and the ability to adapt well to the quarantine and lockdown measures, even if homeless individuals experienced few social interactions during the pandemic. Thus, we speculatively assumed that their loneliness levels only slightly changed. However, future research is required to test our assumptions.

In the context of the beginning of COVID-19 pandemic in 2020, the frequency of loneliness, as defined by the UCLA3 sum score of ≥ 6, among homeless individuals in Hamburg was 48.5% [22]. An increased rate of loneliness following the start of the lockdown measures, estimated by the UCLA scale, was also observed in France, among 37% of the study participants (May–June 2020) [23]. A comparison of the Hamburg survey of homeless individuals with our present study conducted at a later stage of the pandemic shows a slight decline over time. Comparing these data, it must be considered that their acquisition took place in different regions and at different time points, which leads to the impacts of varying governmental measures (such as lockdown regulations) and may lead to an altered reduction in social and physical contact. This, in turn, could contribute to increased loneliness [39,40]. Therefore, it should be taken into account that no partial lockdown took place at the time of the data collection of our multicentric study. The correlates of loneliness may vary between homeless men and women. Therefore, we investigated them further.

We found higher frequency rates of loneliness among homeless women (47.8%) than in men. The fact that the female gender was more affected by the feeling of loneliness during the pandemic is also evident among the general population, but also among homeless women in other countries [41]. Women experiencing homelessness have poorer outcomes in a number of areas, including mental health and emotional wellbeing [42], and they often withdraw from social networks, feel stigmatized and have difficulty relying on friends for protection and support [41,43].

In the total sample, 57.1% of individuals who had experienced homelessness for less than one month reported being lonely in our study. Furthermore, regression analyses were conducted to examine the factors associated with loneliness, stratified by sex. The regressions showed that, in men, an increased likelihood of loneliness was associated with greater concerns about COVID-19 and a shorter duration of homelessness, whereas in women, a higher likelihood of loneliness was associated with older age (40 to 49 years compared to an age from 18 to 29 years), a lower duration of homelessness, being born in Germany and existing psychiatric problems.

Regarding the regression analyses, we also found—at first, unexpectedly—a positive association between a short duration of homelessness and loneliness. At first glance, one may assume that loneliness increases over time among homeless individuals. Possibly, this could be explained by habituation to the conditions of homelessness over time [39]. In addition, it could be hypothesized that those who have been homeless for a longer period of time may have learned more about available services (social support) and have better skills in order to deal with their survival needs [44] and form friendships with other homeless people and, thus, they may feel less lonely. Therefore, the first weeks of homelessness are a special risk factor for experiencing loneliness, as many daily aspects of life have already changed by this time, but the person has not had enough time to adapt their social behavior.

We also found that existing mental problems were associated with a higher likelihood of loneliness among women. Our findings are in line with a study of homeless women in Spain [45]. This is plausible, because it has been shown that problems associated with mental illness can increase social isolation [46,47]. It must be emphasized, as outlined above, that loneliness, perceived social isolation and objective social isolation are interrelated but do not measure up as the same [18]. For example, individuals can feel lonely without feeling socially isolated. Other studies also suggest a link whereby loneliness and social isolation can independently and jointly affect health through their effects on health-related behaviors. Similar findings were obtained in a large study of older people [48]. According to previous research, women are particularly vulnerable to the negative mental health effects of the COVID-19 pandemic [23,40,49].

However, due to the small number of women included in the given study, these findings should be interpreted with great caution. In our study, a higher likelihood of loneliness was associated with COVID-19-related anxiety in men. Other studies of the general population reported comparable findings [50]. We can assume that such a fear can be burdensome and may lead to further isolation and avoidance of contact with others.

Some strengths and limitations of the study must be acknowledged. Among the overall sample, the proportion of women (*n* = 118) was rather small compared to male individuals. Thus, the results for women should be interpreted with caution. In addition, individuals from this particularly vulnerable population of homeless people are difficult to reach regardless of location. Thus, our study included only homeless individuals who use social services. Therefore, it may be difficult to generalize our findings to homeless individuals who do not use such services. Furthermore, a significant number of missing values should be acknowledged. We used listwise deletion to address missing values. Future research could use more elaborated techniques, such as full information maximum likelihood in logistic regressions (when upcoming Stata versions support this option). In contrast, some strengths should be noted. Vulnerable groups such as homeless individuals have rarely been investigated, especially homeless women. Our multicenter study included 39 facilities and, thus, many homeless individuals (*n*= 634), contributing to our current knowledge of loneliness among homeless individuals in a very unique timeframe such as the pandemic.

In addition, it should be noted that our study did not include data on COVID-19 progression. It is important to also further investigate COVID-19 sufferers and their treatment/medical support and to report the associations between these COVID-related health factors and their loneliness. Thus, future research is needed to examine whether such factors are associated with loneliness.

Moreover, an established tool was used to quantify loneliness and was enrolled within our multicenter study. Additionally, we used a dichotomous question to quantify mental health. This has some shortcomings (e.g., it was not possible to distinguish between depression and anxiety). Thus, future research is required to determine the association between mental health and loneliness among homeless individuals in greater detail.

## 5. Conclusions

The COVID-19 pandemic has brought the issue of loneliness to the forefront of research and highlighted its significance. Never before have so many people been affected by loneliness at the same time. It has also raised awareness of the consequences that can be caused by loneliness, showing that preventing loneliness contributes not only to the general wellbeing of the individual, but also of the population, and that it can also reduce the burden on the healthcare system (by maintaining health via the prevention of loneliness). Loneliness, therefore, also constitutes a financial burden on the healthcare system. Thus, ways of preventing and combating loneliness should be discussed more openly.

In summary, homeless individuals experienced the effects of loneliness more intensely than their peers in the general population during the COVID-19 pandemic. Our study results highlight the associations between some explanatory variables, such as the duration of homelessness or mental health problems and the feeling of loneliness among homeless individuals.

Knowledge of the factors associated with high levels of loneliness may help us to identify homeless individuals at risk of loneliness. However, future research in other countries and further longitudinal studies are required to confirm our findings presented here.

## Figures and Tables

**Table 1:** Frequency rates of loneliness (total sample stratified by sex).

	*n*	Loneliness Total Sample	*p*-Value	Men *n*	Loneliness Men	*p*-Value	Women *n*	Loneliness Women	*p*-Value
Overall	265	41.7%		211	40.7%		54	47.8%	
Age (years)			0.85			0.78			0.35
18–29	33	39.3%		29	42.7%		4	25.0%	
30–39	64	39.8%		48	37.2%		16	51.6%	
40–49	72	45.6%		56	44.8%		16	50.0%	
50–59	60	40.5%		47	37.3%		13	48.2%	
>60	23	39.7%		19	40.4%		4	66.7%	
Marital status			0.24			0.09			0.60
married	7	26.9%		5	23.8%		2	40.0%	
married, living apart	20	44.4%		15	42.9%		5	50.0%	
single	180	43.8%		156	44.1%		24	44.4%	
widowed	8	47.1%		2	22.2%		6	75.0%	
divorced	39	34.8%		26	31.3%		13	46.4%	
Children			0.28			0.18			0.80
no	143	43.6%		122	43.1%		21	48.8%	
yes	115	39.1%		83	37.2%		32	46.4%	
Level of education			0.91			0.64			0.57
no school education	43	39.4%		31	37.4%		12	48.0%	
school education	116	41.4%		95	41.7%		21	41.2%	
vocational education	76	43.4%		64	42.7%		12	50.0%	
higher tertiary education	18	39.1%		11	35.4%		7	63.6%	
Home country			<0.01			< 0.05			0.25
other than Germany	108	36.2%		91	35.7%		16	40.0%	
Germany	151	47.0%		114	46.3%		37	51.4%	
Duration of homelessness (months)			<0.01			0.14			<0.05
1 month	28	57.1%		19	51.4%		9	75.0%	
2–12 months	79	35.0%		66	36.3%		13	31.0%	
>13 months	147	45.0%		117	43.5%		30	53.6%	
Having a pet			0.27			0.43			0.32
no	238	42.0%		191	41.1%		47	48.5%	
yes	17	34.0%		13	34.2%		4	33.3%	
Alcohol consumption			<0.05			0.07			0.10
never	82	39.2%		60	37.3%		22	46.8%	
once or twice a year	32	47.8%		26	50.0%		6	42.9%	
monthly	37	44.6%		27	41.5%		9	56.3%	
weekly	20	25.6%		18	29.5%		2	15.4%	
daily	86	46.7%		73	44.8%		13	61.9%	
Illegal drugs			0.44			0.36			0.97
never	143	38.9%		111	37.5%		31	47.7%	
once or twice a year	12	54.5%		11	55.0%		1	50.0%	
monthly	20	40.0%		18	40.9%		2	33.3%	
weekly	21	45.7%		17	43.6%		3	50.0%	
daily	62	45.6%		48	45.7%		14	46.7%	
Mental health concerns			<0.001			<0.01			<0.01
no	178	36.9%		148	36.9%		28	37.8%	
yes	81	56.3%		56	53.3%		25	65.8%	
Concerns about COVID-19		1.7 (1.0)	<0.001		1.6 (1.0)	<0.01		1.8 (1.1)	

**Table 2:** Predictors of loneliness stratified by sex. Results of multiple logistic regression.

	Loneliness	
Variables	Men	Women
Age group (reference group: 18 to 29 years)		
>30–39 years	0.55	9.02
	(0.27–1.15)	(0.32–253.29)
>40–49 years	0.95	37.43 *
	(0.46–1.98)	(1.58–885.63)
>50–59 years	0.82	3.00
	(0.38–1.78)	(0.14–65.61)
>60 years	0.77	0.76
	(0.30–1.98)	(0.01–59.20)
Marital status (reference group: married)		
married, living apart	1.56	6.03
	(0.36–6.87)	(0.11–342.87)
single	1.66	1.47
	(0.45–6.07)	(0.04–61.21)
widowed	0.34	20.53
	(0.04–2.70)	(0.25–1667.98)
divorced	1.27	1.04
	(0.32–5.03)	(0.02–43.41)
Having children	1.00	1.89
(reference group: not having children) Education (reference group: no school education)	(0.63–1.61)	(0.34–10.35)
school education	1.10	0.30
	(0.59–2.08)	(0.05–1.77)
vocational education	1.33	1.13
	(0.68–2.61)	(0.16–7.80)
higher tertiary education	0.97	1.35
Country of origin	(0.36–2.61)	(0.10–19.19)
(reference group: other than Germany) Duration of homelessness (reference group: up to 1 month)	1.06 (0.67–1.67)	8.57 * (1.11–66.52)
>2–12 months	0.38 *	0.02 **
	(0.16–0.91)	(0.00–0.26)
>12 months	0.52	0.07 *
	(0.22–1.23)	(0.01–0.87)
Pets	0.50	0.09 +
(reference group: having no pets)	(0.20–1.22)	(0.01–1.56)
Alcohol consumption (reference group: no alcohol consumption)		
once or twice a year	1.52	1.80
	(0.70–3.27)	(0.23–13.95)
monthly	0.94	6.66
	(0.46–1.93)	(0.58–76.60)
weekly	0.64	0.04 **
	(0.30–1.36)	(0.00–0.40)
daily	1.27	0.66
Consumption of illegal drugs (reference group: no illegal drug use)	(0.74–2.20)	(0.09–4.87)
once or twice a year	2.53	-
	(0.78–8.23)	-
monthly	1.28	0.61
	(0.59–2.79)	(0.01–25.47)
weekly	1.04	0.22
daily	(0.45–2.40) 1.08 (0.61–1.93)	(0.01–6.56) 0.26 (0.03–2.11)
Mental health concerns	1.59 +	5.89 *
(reference group: absence of mental health problems)	(0.95–2.65)	(1.31–26.39)
Concerns about COVID-19	1.34 * (1.06–1.70)	1.44 (0.71–2.92)
Constant	0.64	0.04
	(0.08–5.37)	(0.00–34.17)
Observations	405	91
(McFadden’s) Pseudo R²	0.06	0.40

Odds ratios are reported; 95% CI in parentheses; ** *p* < 0.01, * *p* < 0.05, + *p* < 0.10.

## Data Availability

The datasets analyzed during the current study are not publicly available due to ethical restrictions involving patient data but are available from the corresponding author on reasonable request.

## Supplementary Materials

The following supporting information can be downloaded at: https://www.mdpi.com/article/10.3390/ijerph191912718/s1, Supplementary Table S1: Correlation matrix for continuous measures (among total sample); Supplementary Table S2: Correlation matrix for continuous measures (among men). Supplementary Table S3: Correlation matrix for continuous measures (among women).

## References

[1] Federal Association for Assistance to Homeless People (BAGW): Zahl der Wohnungslosen_Number of Homeless Individuals. https://www.bagw.de/de/themen/zahl-der-wohnungslosen/index.html.

[2] Ralli M., Morrone A., Arcangeli A., Ercoli L. (2021). Asymptomatic patients as a source of transmission of COVID-19 in homeless shelters. Int. J. Infect. Dis..

[3] Tobolowsky F.A., Gonzales E., Self J.L., Rao C.Y., Keating R., Marx G.E., McMichael T.M., Lukoff M.D., Duchin J.S., Huster K. (2020). COVID-19 Outbreak Among Three Affiliated Homeless Service Sites—King County, Washington, 2020. MMWR Morb. Mortal. Wkly. Rep..

[4] Tsai J., Wilson M. (2020). COVID-19: A potential public health problem for homeless populations. Lancet Public Health.

[5] Wood L.J., Davies A.P., Khan Z. (2020). COVID-19 precautions: Easier said than done when patients are homeless. Med. J. Aust..

[6] Schindel D., Kleyer C., Schenk L. (2020). Somatic diseases of homeless people in Germany. A narrative literature review for the years 2009–2019. Bundesgesundheitsblatt Gesundh. Gesundh..

[7] Schrooyen L., Delforge M., Lebout F., Vanbaelen T., Lecompte A., Dauby N. (2021). Homeless people hospitalized with COVID-19 in Brussels. Clin. Microbiol. Infect..

[8] Perlman D., Peplau L.A. (1981). Toward a social psychology of loneliness. Pers. Relatsh..

[9] Hajek A., König H.H. (2021). Social Isolation and Loneliness of Older Adults in Times of the COVID-19 Pandemic: Can Use of Online Social Media Sites and Video Chats Assist in Mitigating Social Isolation and Loneliness?. Gerontology.

[10] Cacioppo J.T., Hawkley L.C. (2009). Perceived social isolation and cognition. Trends Cogn. Sci..

[11] Dahlberg L. (2021). Loneliness during the COVID-19 pandemic. Aging Ment. Health.

[12] Hajek A., Kretzler B., König H.H. (2021). Informal Caregiving, Loneliness and Social Isolation: A Systematic Review. Int. J. Environ. Res. Public Health.

[13] Das A., Padala K.P., Crawford C.G., Teo A., Mendez D.M., Phillips O.A., Wright B.C., House S., Padala P.R. (2021). A systematic review of loneliness and social isolation scales used in epidemics and pandemics. Psychiatry Res..

[14] Maes M., Qualter P., Vanhalst J., Van den Noortgate W., Goossens L. (2019). Gender Differences in Loneliness across the Lifespan: A Meta–Analysis. Eur. J. Personal..

[15] Takagi E., Saito Y., Chan A. (2020). Gender differences in the association between social relationships and loneliness among older adults in Singapore. J. Popul. Res..

[16] McQuaid R.J., Cox S.M.L., Ogunlana A., Jaworska N. (2021). The burden of loneliness: Implications of the social determinants of health during COVID-19. Psychiatry Res..

[17] European C., Joint Research C., Cassio L., d’Hombres B., Tintori G., Baarck J., Pásztor Z., Balahur A. (2021). Loneliness in the EU: Insights from Surveys and Online Media Data.

[18] Hajek A., König H.H. (2022). Prevalence and correlates of loneliness, perceived and objective social isolation during the COVID-19 pandemic. Evidence from a representative survey in Germany. Soc. Psychiatry Psychiatr. Epidemiol..

[19] Varga T.V., Bu F., Dissing A.S., Elsenburg L.K., Bustamante J.J.H., Matta J., van Zon S.K.R., Brouwer S., Bültmann U., Fancourt D. (2021). Loneliness, worries, anxiety, and precautionary behaviours in response to the COVID-19 pandemic: A longitudinal analysis of 200,000 Western and Northern Europeans. Lancet Reg. Health Eur..

[20] Harden K., Price D.M., Mason H., Bigelow A. (2020). COVID-19 Shines a Spotlight on the Age-Old Problem of Social Isolation. J. Hosp. Palliat. Nurs..

[21] Rumas R., Shamblaw A.L., Jagtap S., Best M.W. (2021). Predictors and consequences of loneliness during the COVID-19 Pandemic. Psychiatry Res..

[22] Bertram F., Heinrich F., Fröb D., Wulff B., Ondruschka B., Püschel K., König H.H., Hajek A. (2021). Loneliness among Homeless Individuals during the First Wave of the COVID-19 Pandemic. Int. J. Environ. Res. Public Health.

[23] Scarlett H., Davisse-Paturet C., Longchamps C., Aarbaoui T.E., Allaire C., Colleville A.C., Convence-Arulthas M., Crouzet L., Ducarroz S., Melchior M. (2021). Depression during the COVID-19 pandemic amongst residents of homeless shelters in France. J. Affect. Disord. Rep..

[24] Zaman R., Hankir A., Jemni M. (2019). Lifestyle Factors and Mental Health. Psychiatr. Danub..

[25] Cacioppo S., Grippo A.J., London S., Goossens L., Cacioppo J.T. (2015). Loneliness: Clinical import and interventions. Perspect. Psychol. Sci..

[26] Bundesregierung/Corona-Regelungen. https://www.bundesregierung.de/breg-de/aktuelles/bund-laender-beratung-corona-1949606.

[27] Robert Koch Institut_Situationsbericht. https://www.rki.de/DE/Content/InfAZ/N/Neuartiges_Coronavirus/Situationsberichte/Aug_2021/2021-08-20-de.pdf?__blob=publicationFile.

[28] Russell D.W. (1996). UCLA Loneliness Scale (Version 3): Reliability, validity, and factor structure. J. Pers. Assess..

[29] Ge L., Yap C.W., Ong R., Heng B.H. (2017). Social isolation, loneliness and their relationships with depressive symptoms: A population-based study. PLoS ONE.

[30] Domènech-Abella J., Lara E., Rubio-Valera M., Olaya B., Moneta M.V., Rico-Uribe L.A., Ayuso-Mateos J.L., Mundó J., Haro J.M. (2017). Loneliness and depression in the elderly: The role of social network. Soc. Psychiatry Psychiatr. Epidemiol..

[31] Dahlberg L., McKee K.J., Frank A., Naseer M. (2022). A systematic review of longitudinal risk factors for loneliness in older adults. Aging Ment. Health.

[32] Kretzler B., König H.H., Hajek A. (2022). Pet ownership, loneliness, and social isolation: A systematic review. Soc. Psychiatry Psychiatr. Epidemiol..

[33] Kraav S.L., Awoyemi O., Junttila N., Vornanen R., Kauhanen J., Toikko T., Lehto S.M., Hantunen S., Tolmunen T. (2021). The effects of loneliness and social isolation on all-cause, injury, cancer, and CVD mortality in a cohort of middle-aged Finnish men. A prospective study. Aging Ment. Health.

[34] Van As B.A.L., Imbimbo E., Franceschi A., Menesini E., Nocentini A. (2022). The longitudinal association between loneliness and depressive symptoms in the elderly: A systematic review. Int. Psychogeriatr..

[35] Ejiri M., Kawai H., Ishii K., Oka K., Obuchi S. (2021). Predictors of older adults‘ objectively measured social isolation: A systematic review of observational studies. Arch. Gerontol. Geriatr..

[36] Hansen T., Nilsen T.S., Yu B., Knapstad M., Skogen J.C., Vedaa Ø., Nes R.B. (2021). Locked and lonely? A longitudinal assessment of loneliness before and during the COVID-19 pandemic in Norway. Scand. J. Public Health.

[37] Stickley A., Ueda M. (2022). Loneliness in Japan during the COVID-19 pandemic: Prevalence, correlates and association with mental health. Psychiatry Res..

[38] Killgore W.D.S., Cloonan S.A., Taylor E.C., Dailey N.S. (2020). Loneliness: A signature mental health concern in the era of COVID-19. Psychiatry Res..

[39] Mata J., Wenz A., Rettig T., Reifenscheid M., Möhring K., Krieger U., Friedel S., Fikel M., Cornesse C., Blom A.G. (2021). Health behaviors and mental health during the COVID-19 pandemic: A longitudinal population-based survey in Germany. Soc. Sci. Med..

[40] Bu F., Steptoe A., Fancourt D. (2020). Loneliness during a strict lockdown: Trajectories and predictors during the COVID-19 pandemic in 38,217 United Kingdom adults. Soc. Sci. Med..

[41] Vázquez J.J., Panadero S., Pascual I. (2019). The Particularly Vulnerable Situation of Women Living Homeless in Madrid (Spain). Span. J. Psychol..

[42] Duke A., Searby A. (2019). Mental Ill Health in Homeless Women: A Review. Issues Ment. Health Nurs..

[43] Beutel M.E., Hettich N., Ernst M., Schmutzer G., Tibubos A.N., Braehler E. (2021). Mental health and loneliness in the German general population during the COVID-19 pandemic compared to a representative pre-pandemic assessment. Sci. Rep..

[44] Lewis J.H., Andersen R.M., Gelberg L. (2003). Health care for homeless women. J. Gen. Intern. Med..

[45] Rodriguez-Moreno S., Panadero S., Vázquez J.J. (2020). Risk of mental ill-health among homeless women in Madrid (Spain). Arch. Women’s Ment. Health.

[46] Costa M., Pavlo A., Reis G., Ponte K., Davidson L. (2020). COVID-19 Concerns Among Persons With Mental Illness. Psychiatr. Serv..

[47] Heron P., Spanakis P., Crosland S., Johnston G., Newbronner E., Wadman R., Walker L., Gilbody S., Peckham E. (2022). Loneliness among people with severe mental illness during the COVID-19 pandemic: Results from a linked UK population cohort study. PLoS ONE.

[48] Shankar A., McMunn A., Banks J., Steptoe A. (2011). Loneliness, social isolation, and behavioral and biological health indicators in older adults. Health Psychol..

[49] Almeida M., Shrestha A.D., Stojanac D., Miller L.J. (2020). The impact of the COVID-19 pandemic on women’s mental health. Arch. Women’s Ment. Health.

[50] Jaspal R., Breakwell G.M. (2022). Socio-economic inequalities in social network, loneliness and mental health during the COVID-19 pandemic. Int. J. Soc. Psychiatry.

