# Supplementary material for: Predictors of Loneliness among Homeless Individuals in Germany during the COVID-19 Pandemic
Source: Int J Environ Res Public Health. 2022 Oct 5;19(19):12718. doi: 10.3390/ijerph191912718 (PMC9566392; doi:10.3390/ijerph191912718)
Supplement: Supplementary file 1 [file ijerph-19-12718-s001.zip › ijerph-1865637-supplementary.pdf]

Supplementary Table S1. Correlation matrix for continuous measures (among total sample).

	1	2	3
1: Age	1		
2: Concerning of COVID-19	0.2***	1	
3: Loneliness	0.0+	0.1**	1

*** p<0.001, ** p<0.01, + p<0.10

Supplementary Table S2. Correlation matrix for continuous measures (among men).

	1	2	3
1: Age	1		
2: Concerning of COVID-19	0.2+	1	
3: Loneliness	-0.0+	0.1+	1

+ p<0.10

Supplementary Table S3. Correlation matrix for continuous measures (among women).

	1	2	3
1: Age	1		
2: Concerning of COVID-19	0.2*	1	
3: Loneliness	0.2*	0.2*	1

* p<0.05
